# Supplementary material for: Methodologies for 176Lu–176Hf Analysis of Zircon Grains from the Moon and Beyond
Source: ACS Earth Space Chem. 2023 Dec 7;8(1):36–53. doi: 10.1021/acsearthspacechem.3c00093 (PMC10801744; doi:10.1021/acsearthspacechem.3c00093)
Supplement: Supplementary file 1 — sp3c00093_si_001.pdf [file sp3c00093_si_001.pdf]

Supporting Information

Methodologies for ^{176}Lu-^{176}Hf Analysis of Zircon Grains from the Moon and Beyond

Xi Chen[1], Nicolas Dauphas[1*], Zhe J. Zhang[1], Blair Schoene[2], Melanie Barboni[3], Ingo Leya[4], Junjun Zhang[1], Dawid Szymanowski[2], Kevin D. McKeegan[5]

[1]Origins Laboratory, Department of the Geophysical Sciences and Enrico Fermi Institute, The University of Chicago, Chicago, IL 60637, USA

[2]Department of Geosciences, Princeton University, Princeton, NJ 08544, USA

[3]CLAS-NS Departments, Arizona State University, Tempe, AZ 85281, USA

[4]Physics Institute, University of Bern, Sidlerstrasse 5, 3012 Bern, Switzerland

[5]Department of Earth, Planetary, and Space Sciences, University of California, Los Angeles, CA 90095, USA

1. Initial Hf isotopic composition

As discussed in the main text, we express $\varepsilon^{176}\mathrm{Hf}_{\mathrm{zrc}\text{-}t,c}$, $\varepsilon^{176}\mathrm{Hf}_{\mathrm{CHUR}\text{-}t}$, and the difference $\varepsilon^{176}\mathrm{Hf}_{\mathrm{zrc}\text{-}t,c} - \varepsilon^{176}\mathrm{Hf}_{\mathrm{CHUR}\text{-}t}$ as functions (f) of several random variables (x) and a constant (C),

$$\varepsilon^{176}\mathrm{Hf}_{\mathrm{zrc}\text{-}t,c/\widetilde{\mathrm{CHUR}}\text{-t}} = f_2 = \left[\frac{x_1 - x_4(e^{x_5 x_6} - 1)}{C} - 1\right] \times 10^4 - \frac{x_1 x_2 x_3}{C}, \tag{22}$$

$$\varepsilon^{176}\mathrm{Hf}_{\mathrm{CHUR}\text{-}t/\widetilde{\mathrm{CHUR}}\text{-t}} = f_3 = \left[\frac{x_7 + x_8(e^{x_5 x_9} - e^{x_5 x_6})}{C} - 1\right] \times 10^4, \tag{23}$$

$$\varepsilon^{176}\mathrm{Hf}_{\mathrm{zrc}\text{-}t,c/\widetilde{\mathrm{CHUR}}\text{-t}} - \varepsilon^{176}\mathrm{Hf}_{\mathrm{CHUR}\text{-}t/\widetilde{\mathrm{CHUR}}\text{-t}} = f_4 = \frac{x_1 - x_4(e^{x_5 x_6} - 1) - x_7 - x_8(e^{x_5 x_9} - e^{x_5 x_6})}{C} \times 10^4 - \frac{x_1 x_2 x_3}{C}, \tag{24}$$

with C calculated from the mean values of some variables,

$$C = \widetilde{x_7} + \widetilde{x_8}\left(e^{\widetilde{x_5}\widetilde{x_9}} - e^{\widetilde{x_5}\widetilde{x_6}}\right). \quad \text{(S1)}$$

The mean and standard deviation of each variable are given in the main text. Uncertainty propagation is complicated by the fact that some uncertainties are correlated, which can be tackled using the delta method[1].

1.1. Error propagation in function f_2

The formula that gives the variance of f_2 is,

$$\sigma_{f_2}^2 \simeq \nabla g_2 \times V_2 \times \nabla g_2^T, \quad \text{(S2)}$$

where V_2 is the covariance matrix,

$$V_2 = \begin{bmatrix} v(x_1, x_1) & 0 & v(x_1, x_3) & 0 & 0 & 0 \\ 0 & v(x_2, x_2) & 0 & 0 & 0 & 0 \\ v(x_1, x_3) & 0 & v(x_3, x_3) & 0 & 0 & 0 \\ 0 & 0 & 0 & v(x_4, x_4) & 0 & 0 \\ 0 & 0 & 0 & 0 & v(x_5, x_5) & 0 \\ 0 & 0 & 0 & 0 & 0 & v(x_6, x_6) \end{bmatrix}, \quad \text{(S3)}$$

and ∇g_2 is the gradient vector,

$$\nabla g_2 = \begin{bmatrix} \frac{\partial f_2}{\partial x_1} & \cdots & \frac{\partial f_2}{\partial x_6} \end{bmatrix}. \quad \text{(S4)}$$

The only non-zero term in the covariance matrix is $v(x_1, x_3)$, as some dependence exists between $^{176}Hf/^{177}Hf$ and $^{178,180}Hf/^{177}Hf$ ratios through sharing of common isotopes in the internal normalization scheme. To calculate this correlation coefficient, we again use the delta method on the bracketing-standard normalization. The internally normalized $^{176}\mathrm{Hf}/^{177}\mathrm{Hf}$ ratio of a zircon (S_1) is normalized to the two measured ratios of bracketing standards S_2 and S_3, and their known absolute $^{176}\mathrm{Hf}/^{177}\mathrm{Hf}$ ratio: s through,

$$x_1 = \frac{2sS_1}{S_2 + S_3}. \quad \text{(S5)}$$

We take s as 0.282160 for $^{176}Hf/^{177}Hf$ ratio of JMC-475 Hf standard in this study. Similarly, the measured ε^iHf values of zircons were calculated from internally normalized

$\left({}^{i}\mathrm{Hf}/{}^{177}\mathrm{Hf}\right)_{\text{zrc-p}}$ ratios (R; $i = 178$ or 180), which are also bracketed by two internally normalized bracketing standards (R_2 and R_3),

$$x_3 = 10^4\left(\frac{2R_1}{R_2+R_3} - 1\right). \quad \text{(S6)}$$

We note g as the function that calculates $^{176}Hf/^{177}Hf$ (x_1) and $\varepsilon^{178/180}Hf$ (x_3) normalized by standard bracketing from the internally normalized ratio $(S_1, S_2, S_3, R_1, R_2, R_3)$,

$$g(S_1, S_2, S_3, R_1, R_2, R_3) = (x_1, x_3). \quad \text{(S7)}$$

The gradient matrix for (x_1, x_3) can be written as,

$$\nabla g = \begin{bmatrix} \frac{2s}{S_2+S_3} & \frac{-2sS_1}{(S_2+S_3)^2} & \frac{-2sS_1}{(S_2+S_3)^2} & 0 & 0 & 0 \\ 0 & 0 & 0 & \frac{10^4}{(R_2+R_3)} & \frac{-2\times10^4 R_1}{(R_2+R_3)^2} & \frac{-2\times10^4 R_1}{(R_2+R_3)^2} \end{bmatrix}. \quad \text{(S8)}$$

The covariance matrix of $(S_1, S_2, S_3, R_1, R_2, R_3)$ takes the form,

$$\epsilon = \begin{bmatrix} cov(S_1,S_1) & 0 & 0 & cov(S_1,R_1) & 0 & 0 \\ 0 & cov(S_2,S_2) & 0 & 0 & cov(S_2,R_2) & 0 \\ 0 & 0 & cov(S_3,S_3) & 0 & 0 & cov(S_3,R_3) \\ cov(S_1,R_1) & 0 & 0 & cov(R_1,R_1) & 0 & 0 \\ 0 & cov(S_2,R_2) & 0 & 0 & cov(R_2,R_2) & 0 \\ 0 & 0 & cov(S_3,R_3) & 0 & 0 & cov(R_3,R_3) \end{bmatrix}.$$

(S9)

The null entries in this matrix stem from the fact that measurements made at different times are expected to be independent. The covariance matrix for x_1 and x_3 is,

$$v(x_1, x_3) = \begin{bmatrix} \sigma_1\sigma_1 & \rho\sigma_1\sigma_3 \\ \rho\sigma_1\sigma_3 & \sigma_3\sigma_3 \end{bmatrix}. \quad \text{(S10)}$$

According to the delta method, the covariance matrix can be approximated by,

$$v(x_1, x_3) = \begin{bmatrix} cov(x_1,x_1) & cov(x_1,x_3) \\ cov(x_1,x_3) & cov(x_3,x_3) \end{bmatrix} = \nabla g \cdot \epsilon \cdot \nabla g^{\mathrm{T}}, \quad \text{(S11)}$$

This can then be injected in Eq. S11 to S2 and we have,

$$\sigma_{f_2}^2 \simeq \left(\frac{10^4}{C}\right)^2 \left[\left(1-\frac{x_2x_3}{10^4}\right)^2 \sigma_{x_1}^2 + (e^{x_5x_6}-1)^2\sigma_{x_4}^2 + (x_4x_6e^{x_5x_6})^2\sigma_{x_5}^2 + (x_4x_5e^{x_5x_6})^2\sigma_{x_6}^2\right] +$$

$$\frac{x_1^2x_3^2}{C^2}\sigma_{x_2}^2 + \frac{x_1^2x_2^2}{C^2}\sigma_{x_3}^2 + 2\frac{10^4}{C^2}\left(1-\frac{x_2x_3}{10^4}\right)x_1x_2\rho\sigma_{x_1x_3}, \quad \text{(S12)}$$

The correlation coefficients (ρ) are calculated based on the measured $^{176}Hf/^{177}Hf$ and $^{178/180}Hf/^{177}Hf$, and they are given in **Table S1**.

1.2. Error propagation in function f_3

Most of the parameters in f_3 are clearly independent. The solar system initial $\left(^{176}\mathrm{Hf}/^{177}\mathrm{Hf}\right)_{\text{CHUR-ss}}$ ratio (x_7) from Iizuka et al. (2015) is calculated using Lu-Hf isotopes and Pb-Pb ages from eucrite zircons. The present $(^{176}\mathrm{Lu}/^{177}\mathrm{Hf})_{\text{CHUR-}p}$ was derived from chondrite measurements (x_5), while the solar system ages (x_9) were independently constrained by Pb-Pb dating[2]. The covariance matrix for function f_3 therefore takes the form,

$$V_3 = \begin{bmatrix} v(x_5,x_5) & \cdots & 0 \\ \vdots & \ddots & \vdots \\ 0 & \cdots & v(x_9,x_9) \end{bmatrix}. \quad \text{(S13)}$$

The gradient vector is,

$$\nabla g_3 = \left[\frac{\partial f_3}{\partial x_5} \quad \cdots \quad \frac{\partial f_3}{\partial x_8}\right]. \quad \text{(S14)}$$

The uncertainty of f_3 is calculated using the delta method,

$$\sigma_{f_3}^2 \simeq \nabla g_3 \times V_3 \times \nabla g_3^T. \quad \text{(S15)}$$

and we have,

$$\sigma_{f_3}^2 \simeq \left(\frac{10^4}{C}\right)^2 \left[(x_8x_9e^{x_5x_9} - x_8x_6e^{x_5x_6})^2\sigma_{x_5}^2 + (x_5x_8e^{x_5x_6})^2\sigma_{x_6}^2 + \sigma_{x_7}^2 + (e^{x_5x_9} - e^{x_5x_6})^2\sigma_{x_8}^2 + (x_5x_8e^{x_5x_9})^2\sigma_{x_9}^2\right]. \quad \text{(S16)}$$

1.3. Error propagation in function f_4

As in 1.1, we have to deal with the fact that x_1 and x_3 are not independent. The steps used to calculate the uncertainty as in 1.1 and are not repeated here. The uncertainty on $\varepsilon^{176}\mathrm{Hf}_{\mathrm{zrc}\text{-}t,c} - \varepsilon^{176}\mathrm{Hf}_{\mathrm{CHUR}\text{-}t}$ (f_4) can be calculated as,

$$\sigma_{f_4}^2 \simeq \nabla g_4 \times V_4 \times \nabla g_4^T. \tag{S17}$$

with V_4 as the covariance matrix and ∇g_4 as the gradient vectors. We therefore have,

$$\sigma_{f_4}^2 \simeq \left(\frac{10^4}{C}\right)^2 \left[\left(1-\frac{x_2 x_3}{10^4}\right)^2 \sigma_{x_1}^2 + (e^{x_5 x_6}-1)^2 \sigma_{x_4}^2 + (x_4 x_6 e^{x_5 x_6} + x_8 x_9 e^{x_5 x_9} - x_8 x_6 e^{x_5 x_6})^2 \sigma_{x_5}^2 + (-x_4 x_5 e^{x_5 x_6} + x_5 x_8 e^{x_5 x_6})^2 \sigma_{x_6}^2 + \sigma_{x_7}^2 + (e^{x_5 x_9} - e^{x_5 x_6})^2 \sigma_{x_8}^2 + (x_5 x_8 e^{x_5 x_9})^2 \sigma_{x_9}^2\right] + \frac{x_1^2 x_3^2}{c^2}\sigma_{x_2}^2 + \frac{x_1^2 x_2^2}{c^2}\sigma_{x_3}^2 + 2\frac{10^4}{C^2}\left(1-\frac{x_2 x_3}{10^4}\right) x_1 x_2 \sigma_{x_1 x_3}. \tag{S18}$$

For each zircon, we have calculated the contribution of each variable $\left(\frac{\partial f_3}{\partial x_i}\right)^2 \sigma_{x_i}^2/\sigma_{f_3}^2$ and $2\frac{(\partial f_3)^2}{\partial x_1 \partial x_3}\sigma_{x_{1,3}}^2/\sigma_{f_3}^2$ to the overall variance of f_4 (**Table S1**). The main sources of error in $\varepsilon^{176}\mathrm{Hf}_{\mathrm{zrc}\text{-}t,c} - \varepsilon^{176}\mathrm{Hf}_{\mathrm{CHUR}\text{-}t}$ are the measured $(^{176}\mathrm{Hf}/^{177}\mathrm{Hf})_{\mathrm{zrc}\text{-}p}$ ratio (x_1), the measured isotopic shifts $\varepsilon^{178}\mathrm{Hf}$ and $\varepsilon^{180}\mathrm{Hf}$ that are used to correct cosmogenic effects (x_3), and the initial Hf isotopic composition of CHUR $(^{176}\mathrm{Hf}/^{177}\mathrm{Hf})_{\mathrm{CHUR}\text{-}ss}$ (x_7). Uncertainties from x_2,x_4,x_5,x_6,x_8, and x_9 are entirely negligible because they are either small or cancel out when we calculate the difference with CHUR. Neglecting those terms, we have a simpler expression for the error of f_4,

$$\sigma_{f_4}^2 \simeq \left(\frac{10^4}{C}\right)^2 \left[\left(1-\frac{x_2 x_3}{10^4}\right)^2 \sigma_{x_1}^2 + \sigma_{x_7}^2\right] + \frac{x_1^2 x_2^2}{c^2}\sigma_{x_3}^2 - 2\frac{10^4}{C^2}\left(1-\frac{x_2 x_3}{10^4}\right) x_1 x_2 \sigma_{x_1 x_3} \quad .$$

(S19)

2. Model ages

In main text, we used eqn. 29 for the model ages for individual zircon, which in epsilon notation takes the form,

$$e^{\lambda_{^{176}\mathrm{Lu}} t_d} = e^{\lambda_{^{176}\mathrm{Lu}} t} + \frac{C}{10^4}\left[\frac{\varepsilon^{176}\mathrm{Hf}_{\text{zrc-t,c}} - \varepsilon^{176}\mathrm{Hf}_{\text{CHUR-}t}}{(^{176}\mathrm{Lu}/^{177}\mathrm{Hf})_{\text{R-}p} - (^{176}\mathrm{Lu}/^{177}\mathrm{Hf})_{\text{CHUR-}p}}\right]. \quad \text{(S20)}$$

Rearranging the Eq. S20, the model age can be written as:

$$t_d = t + \frac{1}{\lambda_{^{176}Lu}} \ln\left[\frac{\left(\frac{^{176}\mathrm{Hf}}{^{177}\mathrm{Hf}}\right)_{\text{zrc-p}} - \left(\frac{^{176}\mathrm{Lu}}{^{177}\mathrm{Hf}}\right)_{\text{zrc-}p}\left(e^{\lambda_{^{176}\mathrm{Lu}} t} - 1\right) - \left(\frac{^{176}\mathrm{Hf}}{^{177}\mathrm{Hf}}\right)_{\text{CHUR-}ss} - \left(\frac{^{176}\mathrm{Lu}}{^{177}\mathrm{Hf}}\right)_{\text{CHUR-}p}\left(e^{\lambda_{^{176}\mathrm{Lu}} t_{ss}} - e^{\lambda_{^{176}\mathrm{Lu}} t}\right) - \left(\frac{^{176}\mathrm{Hf}}{^{177}\mathrm{Hf}}\right)_{\text{zrc-p}}\left(\frac{\alpha_i \varepsilon^i \mathrm{Hf}}{10^4}\right)}{(^{176}\mathrm{Lu}/^{177}\mathrm{Hf})_{\text{R-}p} - (^{176}\mathrm{Lu}/^{177}\mathrm{Hf})_{\text{CHUR-}p}}\right]. \quad \text{(S21)}$$

Using the previously defined variables, we have,

$$t_d = \frac{1}{x_5} \ln\left[\frac{x_{10} e^{x_5 x_6} + x_1 - x_4(e^{x_5 x_6} - 1) - x_7 - x_8 e^{x_5 x_9} - \frac{x_1 x_2 x_3}{10^4}}{x_{10} - x_8}\right]. \quad \text{(S22)}$$

The standard deviation of t_d can be calculated using the delta method,

$$\sigma_{t_d}^2 \simeq \sum\left(\frac{\partial f}{\partial x_i}\right)^2 \sigma_{x_i}^2 + 2\sum\sum\frac{(\partial f)^2}{\partial x_i \partial x_j}\sigma_{x_i x_j}. \quad \text{(S23)}$$

We now define $g = x_{10} e^{x_5 x_6} + x_1 - x_4(e^{x_5 x_6} - 1) - x_7 - x_8 e^{x_5 x_9} - \frac{x_1 x_2 x_3}{10^4}$, so Eq. S23 takes the form,

$$\sigma_{t_d}^2 \simeq \left[\frac{1}{g x_5}\left(1 - \frac{x_2 x_3}{10^4}\right)\right]^2 \sigma_{x_1}^2 + \left[\frac{1}{g x_5}\left(\frac{x_1 x_3}{10^4}\right)\right]^2 \sigma_{x_2}^2 + \left[\frac{1}{g x_5}\left(\frac{x_1 x_2}{10^4}\right)\right]^2 \sigma_{x_3}^2 + \left[\frac{1}{g x_5}(e^{x_5 x_6} - 1)\right]^2 \sigma_{x_4}^2 + \left[\frac{1}{x_5 g}(x_{10} x_6 e^{x_5 x_6} - x_4 x_6 e^{x_5 x_6} - x_8 x_9 e^{x_5 x_9}) - \frac{1}{x_5^2}\ln(g x_{10} - g x_8)\right]^2 \sigma_{x_5}^2 + \left[\frac{1}{g x_5}(x_{10} x_6 e^{x_5 x_6} - x_4 x_6 e^{x_5 x_6})\right]^2 \sigma_{x_6}^2 + \left(\frac{1}{g x_5}\right)^2 \sigma_{x_7}^2 + \left[\frac{1}{x_5}\left(\frac{1}{(x_{10} - x_8)} - \frac{e^{x_5 x_9}}{g}\right)\right]^2 \sigma_{x_8}^2 + \left(\frac{x_8 x_9 e^{x_5 x_9}}{g x_5}\right)^2 \sigma_{x_9}^2 + \left[\frac{1}{x_5}\left(\frac{(x_{10} - x_8) e^{x_5 x_6}}{g} - \frac{1}{(x_{10} - x_8)}\right)\right]^2 \sigma_{x_{10}}^2 - 2 \times \left[\frac{1}{g x_5}\left(1 - \frac{x_2 x_3}{10^4}\right)\right]\left[\frac{1}{g x_5}\left(\frac{x_1 x_2}{10^4}\right)\right]\sigma_{x_1 x_3}. \quad \text{(S24)}$$

We used this formula to calculate $\sigma_{t_d}^2$ and compared the results with Monte-Carlo simulations, and the two approaches agree (Figure S1). The main sources of errors for the model ages come from

x_1, x_3, and x_7, which together contribute more than 99% to the total error (Table S1). The formulas are incorporated in Table S1.

3. Model ages and initial Lu-Hf ratio

The model age can also be calculated by doing a linear regression of $\varepsilon^{176}\text{Hf}_{\text{zrc-}t,c}(t)$ versus the crystallization age (t) of all or a subset of zircons. In this approach, the intersection between the zircon regression line and CHUR gives the model age and the slope reflects the $(^{176}\text{Lu}/^{177}\text{Hf})_{\text{R-}p}$ of the reservoir R. In the following text, we derive the analytical expression to the $(^{176}\text{Lu}/^{177}\text{Hf})_{\text{R-}p}$ and the model ages.

The $\varepsilon^{176}\text{Hf}_{\text{zrc-t,c}}$ of zircons is a function of crystallization ages (t), and expressed as,

$$\varepsilon^{176}\text{Hf}_{\text{zrc-t,c}} - \varepsilon^{176}\text{Hf}_{\text{CHUR-}t} = \frac{10^4}{C}\left[\left(\frac{^{176}\text{Lu}}{^{177}\text{Hf}}\right)_{\text{R-}p} - \left(\frac{^{176}\text{Lu}}{^{177}\text{Hf}}\right)_{\text{CHUR-}p}\right]\left(e^{\lambda_{^{176}\text{Lu}}t_d} - e^{\lambda_{^{176}\text{Lu}}t}\right). \quad \text{(S24)}$$

The slope of regression line can be expressed as,

$$slope = \frac{10^4}{C}\left[\left(\frac{^{176}\text{Lu}}{^{177}\text{Hf}}\right)_{\text{R-}p} - \left(\frac{^{176}\text{Lu}}{^{177}\text{Hf}}\right)_{\text{CHUR-}p}\right]\frac{\left(e^{\lambda_{^{176}\text{Lu}}t_d} - e^{\lambda_{^{176}\text{Lu}}t}\right)}{t - t_d}. \quad \text{(S25)}$$

Rearranging the equation above, we obtain,

$$\left(\frac{^{176}\text{Lu}}{^{177}\text{Hf}}\right)_{\text{R-}p} = \left(\frac{^{176}\text{Lu}}{^{177}\text{Hf}}\right)_{\text{CHUR-}p} - \frac{C}{10^4}\frac{slope \times (t_d - t)}{\left(e^{\lambda_{^{176}\text{Lu}}t_d} - e^{\lambda_{^{176}\text{Lu}}t}\right)}. \quad \text{(S26)}$$

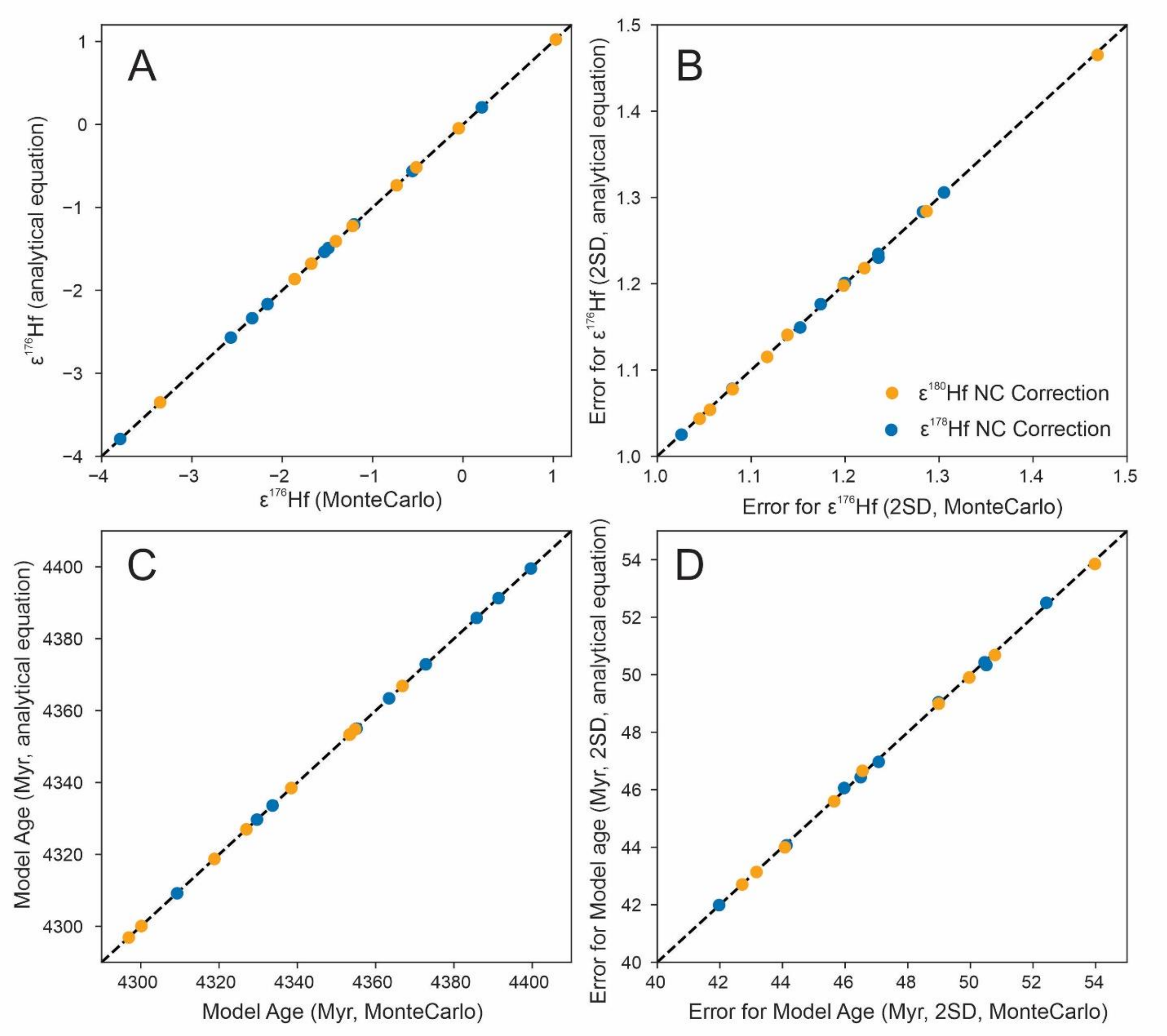

Figure S1. The comparison of calculated ε^{176}Hf (A), Model Ages (C) and their associated errors (B and D) between Monte Carlo simulation and the analytical equations. The two methods yield the same values.

Table S1. Error contributions (%) of each term to the final error of $\varepsilon^{176}Hf_{CHUR\text{-}t}$ and model age of zircon data

Sample	$\left(\frac{\partial f}{\partial x_1}\right)^2 \sigma^2 x_1/\sigma_f^2$		$\left(\frac{\partial f}{\partial x_2}\right)^2 \sigma^2 x_2/\sigma_f^2$		$\left(\frac{\partial f}{\partial x_3}\right)^2 \sigma^2 x_3/\sigma_f^2$		$\left(\frac{\partial f}{\partial x_4}\right)^2 \sigma^2 x_4/\sigma_f^2$		$\left(\frac{\partial f}{\partial x_5}\right)^2 \sigma^2 x_5/\sigma_f^2$		$\left(\frac{\partial f}{\partial x_6}\right)^2 \sigma^2 x_6/\sigma_f^2$		$\left(\frac{\partial f}{\partial x_7}\right)^2 \sigma^2 x_7/\sigma_f^2$		$\left(\frac{\partial f}{\partial x_8}\right)^2 \sigma^2 x_8/\sigma_f^2$		$\left(\frac{\partial f}{\partial x_9}\right)^2 \sigma^2 x_9/\sigma_f^2$		$2\frac{(\partial f_3)^2}{\partial x_1 \partial x_3}\sigma^2_{x_{1,3}}/\sigma^2_{f_3}$	
	**ε^{176}Hf**	t_d	ε^{176}Hf	t_d	ε^{176}Hf	t_d	ε^{176}Hf	t_d	ε^{176}Hf	t_d	ε^{176}Hf	t_d	ε^{176}Hf	t_d	ε^{176}Hf	t_d	ε^{176}Hf	t_d	ε^{176}Hf	t_d
NC-178 correction																				
14163 Z89	57%	57%	1.1%	1.1%	25%	25%	0.6%	0.6%	0.0%	0.1%	0.0%	0.0%	36%	36%	0.0%	0.0%	0.0%	0.0%	-19%	-19%
14163 Z9_L1	43%	43%	0.0%	0.0%	19%	19%	2.9%	2.9%	0.0%	0.1%	0.2%	0.0%	27%	27%	0.0%	0.0%	0.0%	0.0%	7%	7%
14163 Z26_L1	39%	51%	0.3%	0.4%	17%	23%	0.3%	0.3%	0.0%	0.1%	24.5%	0.0%	24%	32%	0.0%	0.0%	0.0%	0.0%	-5%	-7%
14163 Z26_L2	47%	52%	0.3%	0.4%	21%	23%	0.1%	0.1%	0.0%	0.1%	8.3%	0.0%	30%	33%	0.0%	0.0%	0.0%	0.0%	-7%	-8%
14321 Z3_L1	10%	10%	0.0%	0.0%	5%	5%	47%	47%	0.0%	0.2%	0.0%	0.0%	39%	39%	0.1%	0.0%	0.0%	0.0%	-1%	-1%
14321 Z3_L2	16%	16%	0.0%	0.0%	63%	63%	4.3%	4.3%	0.0%	0.1%	0.0%	0.0%	25%	25%	0.0%	0.0%	0.0%	0.0%	-8%	-8%
72275 Z1_L1	18%	18%	0.0%	0.0%	72%	72%	1.1%	1.1%	0.0%	0.1%	0.3%	0.0%	29%	29%	0.0%	0.0%	0.0%	0.0%	-20%	-20%
72275 Z1_L2	17%	17%	0.1%	0.1%	68%	68%	5.6%	5.6%	0.0%	0.1%	0.1%	0.0%	27%	27%	0.0%	0.0%	0.0%	0.0%	-18%	-18%
72275 Z1	50%	50%	0.0%	0.0%	22%	22%	0.5%	0.5%	0.0%	0.1%	0.0%	0.0%	31%	31%	0.0%	0.0%	0.0%	0.0%	-4%	-4%
NC-180 Correction																				
14163 Z89	50%	50%	0.1%	0.1%	63%	63%	1%	1%	0.0%	0.1%	0.0%	0.0%	32%	32%	0.0%	0.0%	0.0%	0.0%	-45%	-45%
14163 Z9_L1	60%	60%	0.0%	0.0%	75%	75%	4%	4%	0.0%	0.2%	0.2%	0.0%	38%	38%	0.0%	0.0%	0.0%	0.0%	-77%	-77%
14163 Z26_L1	31%	38%	0.0%	0.0%	38%	47%	0%	0%	0.0%	0.1%	19.5%	0.0%	19%	24%	0.0%	0.0%	0.0%	0.0%	-7%	-9%
14163 Z26_L2	40%	43%	0.0%	0.0%	49%	53%	0%	0%	0.0%	0.1%	7.0%	0.0%	25%	27%	0.0%	0.0%	0.0%	0.0%	-21%	-23%
14321 Z3_L1	7%	7%	0.0%	0.0%	43%	43%	33%	33%	0.0%	0.1%	0.0%	0.0%	28%	28%	0.0%	0.0%	0.0%	0.0%	-12%	-12%
14321 Z3_L2	24%	24%	0.0%	0.0%	61%	61%	6%	6%	0.0%	0.2%	0.0%	0.0%	37%	37%	0.1%	0.0%	0.0%	0.0%	-28%	-28%
72275 Z1_L1	23%	23%	0.0%	0.0%	58%	58%	1%	1%	0.0%	0.1%	0.4%	0.0%	36%	36%	0.0%	0.0%	0.0%	0.0%	-18%	-18%
72275 Z1_L2	21%	21%	0.0%	0.0%	54%	54%	7%	7%	0.0%	0.1%	0.2%	0.0%	33%	33%	0.0%	0.0%	0.0%	0.0%	-16%	-16%
72275 Z1	46%	46%	0.0%	0.0%	57%	57%	0%	0%	0.0%	0.1%	0.0%	0.0%	29%	29%	0.0%	0.0%	0.0%	0.0%	-32%	-32%

1. Dauphas, N.; Hopp, T.; Craig, G.; Zhang, Z. J.; Valdes, M. C.; Heck, P. R.; Charlier, B. L.; Bell, E. A.; Harrison, T. M.; Davis, A. M., In situ 87 Rb–87 Sr analyses of terrestrial and extraterrestrial samples by LA-MC-ICP-MS/MS with double Wien filter and collision cell technologies. *Journal of Analytical Atomic Spectrometry* **2022,** *37* (11), 2420-2441.
2. Connelly, J. N.; Bizzarro, M.; Krot, A. N.; Nordlund, Å.; Wielandt, D.; Ivanova, M. A., The absolute chronology and thermal processing of solids in the solar protoplanetary disk. *Science* **2012,** *338* (6107), 651-655.
